# Assessing rice yield responses to climate change scenarios using a crop simulation model

**DOI:** 10.7717/peerj.20965

**Published:** 2026-03-12

**Authors:** Alper Baydar

**Affiliations:** Department of Biosystems Engineering, Faculty of Agriculture, Siirt University, Siirt, Türkiye

**Keywords:** DSSAT crop simulation model, Rice, Cultivar genetic coefficients, Climate change, RCP 4.5–8.5

## Abstract

Climate change is considered one of the most significant global environmental challenges of the future, and it is expected to adversely affect crop production. Rice is one of the most widely consumed staple foods in the world. Crop simulation models are tools that help researchers to simulate crop production stages for the future at the selected regions. The aim of this study was to evaluate the accuracy of the DSSAT (Decision Support System for Agrotechnology Transfer) CERES (Crop Environment Resource Synthesis) Rice (DSSAT-CERES-Rice) crop simulation model during its calibration and validation stages, and to assess the impacts of climate change in the Mediterranean region of Türkiye. In the calibration stage, the results showed that the estimated phenological values were within an acceptable range, with an error percentage below 10%. The simulated and observed leaf area index (LAI) showed good agreement with normalized root mean square error (nRMSE) ranging from 17.40% to 28.44% and Willmott’s d-index values of 0.59–0.79. Similarly, biomass simulations were consistent with observations (nRMSE: 5.60–18.52%) with satisfactory d-index values, except under I_1.50_ treatment. Climate change scenarios indicated that average yields under irrigated conditions increased by up to 10% in the late-future period, while rainfed conditions showed decreases of 15–25% due to higher temperatures and shortened growth duration. The findings highlight that the crop simulation model offers a robust framework for evaluating the impacts of climate change and guiding the development of effective adaptation strategies.

## Introduction

Rice is one of the most important agricultural products both in the world agriculture and in Türkiye as it is the most common staple food source for the largest number of people all over the world. A total of 900 thousand tons of rice were produced on 112 thousand ha of land in the 2023–2024 growing season in Türkiye (AEPDI, 2024). However, rice production is highly dependent on irrigation, and the prevalent inefficiencies in water use within rice fields requires the need for more effective and sustainable irrigation management strategies (Liang et al., 2023). The increasing scarcity of irrigation water worldwide poses a significant threat to the sustainable production of rice, necessitating improvements in water use efficiency and adaptive management strategies to ensure long-term food security; also, it is necessary to develop economic water-use technologies and research activities in agricultural areas (Çelik, Baydar & Kayak, 2025). In response to the predicted shortage of water resources under future extreme climate conditions, the efficient management and utilization of limited water supplies has emerged as a critical challenge for sustainable development (Hsieh et al., 2025). In the Mediterranean region, the expected reduction in water resources for agricultural irrigation due to climate change has increased the importance of developing yield-water relationship for water-intensive crops such as rice. Therefore, the use of crop simulation models that incorporate climate, soil, and plant parameters also predict future and account for stress conditions across different crops is becoming increasingly widespread.

According to the IPCC AR6 Synthesis Report, the frequency of heatwaves, intense rainfall, floods, droughts, and compound extremes has risen noticeably, with clear attribution to human-induced warming (IPCC, 2023). In particular, 2024 has been verified as the hottest year on record, showing a global temperature anomaly of nearly +1.55 °C above the pre-industrial baseline, while all years between 2015 and 2024 were listed among the top ten warmest since records began (World Meteorological Organization (WMO), 2025).

The Mediterranean region, widely recognized as a climate-sensitive area, is experiencing warming and drying trends that surpass the global average. Within this zone, Türkiye is expected to be especially vulnerable due to its semi-arid environment and dependence on agriculture. Modeling work by Özdemir et al. (2024) in the Gordes Dam Basin projected that, under RCP 4.5 and RCP 8.5 scenarios, changes in precipitation and temperature during 2031–2060 will significantly influence streamflow, nutrient loads, and agricultural yields. Likewise, Azlak & Şaylan (2024) reported that in Central Anatolia, temperatures will increase markedly under future scenarios, resulting in greater crop water stress. These findings collectively highlight the urgency of adaptation strategies such as climate-resilient crops, efficient irrigation management, and sustainable water-use practices.

In recent years, the integration of field experiments with crop simulation models (CSM) has emerged as a highly effective strategy for optimizing crop management practices. These models, developed through the assimilation of data from agricultural meteorology, soil characteristics, plant physiological processes, and management interventions, are designed to simulate and predict crop growth, development stages, and potential yield outcomes. Crop simulation models not only estimate the potential productivity and morphological traits of plants under varying conditions but also offer timely assessments of vegetation cover dynamics, thus serving as robust tools for real-time decision-making in precision agriculture (Ma et al., 2013).

Most crop simulation models (CSMs) have been designed to support decision-making processes in agricultural land management. Furthermore, these models are capable of simulating optimal growth conditions with non-limiting (water and nitrogen) that are often unattainable in field conditions. This capability makes them particularly valuable for exploring and analyzing potential crop yields under ideal circumstances (Zhang, Zhou & He, 2025).

This study evaluates the performance of the Decision Support System for Agrotechnology Transfer-Crop Environment Resource Synthesis-Rice (DSSAT-CERES-Rice) (Hoogenboom et al., 2024) model for rice production under Mediterranean conditions in Türkiye by calibrating and validating the model using field-observed data, determining optimal cultivar genetic coefficients, and identifying potential limitations associated with model application. In addition, it aims to investigate the impacts of future climate change on rice production by applying three different global climate models (Hadley Centre Global Environment Model version 2 (HadGEM2-ES), Model for Interdisciplinary Research on Climate -Earth System Model (MPI-ESM-MR), and Geophysical Fluid Dynamics Laboratory Earth System Model version 2M (GFDL-ESM2M)) and two representative concentration pathways scenarios (RCP 4.5 and 8.5) across three future periods: near-future (2016–2040), mid-future (2041–2070), and far-future (2071–2098).

## Materials & Methods

### Experimental site and properties

**Figure 1:**
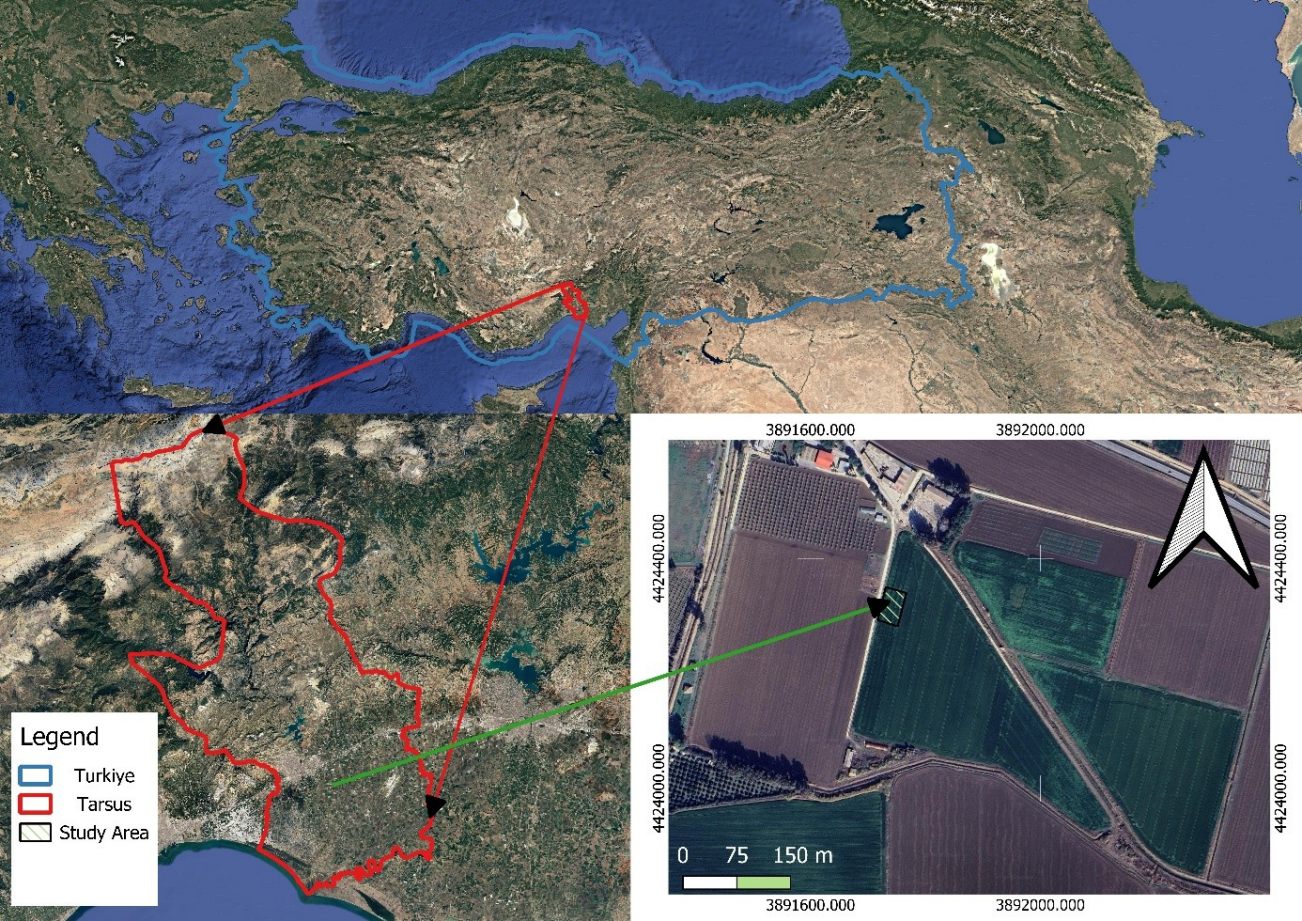
Location of the study site. Satellite image of the experimental site. Map source: https://arastirma.tarimorman.gov.tr/alata.

Research was carried out at the Tarsus location of Alata Horticultural Research Institution (36°53′N and 34°57′E) within 12 m in Mersin, Türkiye in 2019–2020 growing season (Fig. 1) (map background: Google Earth; map data ©Google, ©Maxar Technologies). Climatological conditions at the study area are characteristic of semi-arid of the Mediterranean region in Türkiye with the average annual temperature of 18.2 °C, the average relative humidity is 70.2% and the annual evaporation is 1,478 mm; also, average annual rainfall is 630 mm and mostly distributed from September to May with a high inter-annual variability. Some of physical properties of experimental site were identified by the Bouyoucos hydrometric method (Bouyoucos, 1962) and soil types ranged between clay and silty-clay texture and classified as Arıklı series. The soil water contents at field capacity (FC) and permanent wilting points (WP) are 384 and 273 mm in the 90 cm respectively which were determined by according to (Klute, 1965). The average electrical conductivity (EC) values ranged between 0.40–0.50 dS m^−1^; also, mean bulk density was determined according to standarts as explained by Black et al. (1965) and varied from 1.34 to 1.43 g cm^−3^ with the pH values of 7.8–8.1.

### Treatments and irrigation

The calibration and validation processes are a standard procedure for the performance of crop simulation models. The experimental data collected in 2019 were utilized to calibrate the model for the evaluated rice cultivars, while the independent dataset obtained in 2020 was employed to validate the model performance. For this purpose the field experiment of rice was planted in a randomized split-plot design with three replications consisting of two irrigation methods (DI: surface irrigation and SDI: subsurface drip irrigation) as the main plot and three plant pan coefficients (I_1.00_: Class A-pan evaporation (Ep) x 1.00, I_1.25_: Ep x 1.25 and I_1.50_: Ep x 1.50) treatments as the subplot were considered in the study. The DSSAT model includes only a surface drip irrigation option; therefore, the data from the surface drip irrigation system were considered for all model processes. The experimental subplots had 20 m long and five m wide dimensions (100 m^2^). Irrigations was applied at 2–3 days intervals throughout the growing season based on accumulative evaporation from Class A pan located in the experimental area.

Traditionally, rice is cultivated with continuous flooding throughout all phenological stages except maturity. This cultivation practice accounts for the consumption of over 30 to 45% of the world’s freshwater resources (Parthasarathi et al., 2018). In the field experiment, a drip irrigation system was used. Drippers had 4 L h^−1^ discharge and were applied on every row. Drippers had a wall thickness of one mm and were pressure controlled and spaced on 40 cm. The water resource was a deep well in the experimental site. 90 mm inlet and outlet diameters hydrocyclone and disk filters combinations were used for filtration system and operating pressure of the drip irrigation system was 200 kPa.

### Agronomic practices and cultivar

In this study, a local cultivar of Rekor CL rice (*Oryza sativa* L.) was used and sown with a row spacing of 20 cm on May 24, 2019, and on June 2, 2020, at a seeding rate of 200 kg ha^−^^1^ in both growing season also was harvested manually on October 01, 2019, and October 13, 2020. The fertilizer applications were applied according to soil analysis and the same amounts of total fertilizer were supplied for all treatments. All treatments received 250 kg ha^−1^ diamonium phosphate (DAP, 18-46-0) fertilizer at planting. Ammonium sulphate (21% N) was divided into three equal parts and applied during different growing stages.

### Overview of the DSSAT CERES-rice model

The Decision Support System for Agrotechnology Transfer (DSSAT) is an advanced agricultural modeling platform originally developed within the International Benchmark Sites Network for Agrotechnology Transfer (IBSNAT) program to simulate crop growth, yield, and soil-plant-atmosphere interactions under varying environmental and management conditions.

It enables users to simulate key aspects of crop development, including phenological stages, biomass, yield as well as water and nitrogen dynamics, by utilizing input data related to soil characteristics, climatic conditions, and crop management strategies (Jones et al., 2003; Hoogenboom et al., 2010; Hoogenboom et al., 2019; Sagar et al., 2024). The DSSAT Version 4.8.5.0 was used in this study. The DSSAT model requires daily meteorological inputs such as minimum and maximum air temperatures, solar radiation, wind speed (at two m), relative humidity, and evaporation to run the simulations accurately.

### Data collection and input preparation

The mentioned meteorological data were collected from an automatic station in the study area and entered into the weather management module of the DSSAT-CERES-Rice model. Some of the soil physical (texture, field capacity *etc*.) and chemical (organic carbon, total nitrogen *etc*.) parameters were obtained from soil samples taken from the experimental area in the 0–120 cm depth of the soil and used as inputs to create a soil profile in the model (drained upper and lower limit, saturated soil *etc*.). Soil water content in each plot was determined before irrigation applications with the neutron probe (503 DR) at a depth of 0–120 cm with 30 cm increments during the growing season. Accordingly, the aluminum access tubes were centrally installed in the experimental sub-plots to a depth of 1.5 m. In the crop management section of the model, planting and harvest date, cultivar selection, environmental modifications if needed, irrigation amounts and scheduling, planting methods *etc*. were entered into the model to run.

### Steps for calibrating and validating the DSSAT CERES-rice model

A field experiment was carried out during the 2019–2020 growing season to calibrate and validate the DSSAT-CERES-Rice model. The model was calibrated using phenological data collected from the field experiment, and the genetic coefficients were estimated with the use of the GLUE module integrated into DSSAT. In order to determine the most appropriate genetic coefficients for the study area and rice crop, phenological stages such as anthesis date (ADAT), physiological maturity date (MDAT), emergence date (EDAT), and panicle initiation date (IDAT) were used to calibrate the model. There were three irrigation levels (I_1.00_, I_1.25_ and I_1.50_) considered in the calibration stages, but cultivar genetic coefficients were calculated with I_1.00_ level. For this purpose, these stages were recorded during the field experiment and subsequently entered into the FileA and FileT sections of the experimental data module of the DSSAT model. The validation of the model was performed by comparing the observed field data with the values estimated by the model. For this purpose, yield (kg ha^−^^1^), biomass (kg ha^−^^1^), and leaf area index (m^2^ m^−^^2^) were recorded. Leaf area index (LAI) was measured every two weeks with the aid of a canopy analysis device (LAI-2000). Biomass was assessed using destructive sampling. During each measurement period, plant samples were collected from a designated one m^2^ area in every plot. The samples were then dried in an oven at 70 °C until a constant mass was reached, after which dry biomass was quantified.

### Statistical analysis and model performance evaluation

In the calibration stage, percentage differences (PD) between model-simulated and field-observed data were used. Root mean square error (RMSE), coefficient of determination (R^2^), and the D_index_ were used to evaluate and validate the model. A coefficient of determination (R^2^) value close to 1 reflects a strong agreement between simulated and observed data, indicating that the model effectively captures the variability in the measured values and demonstrates high predictive performance. To understand the discrepancy between estimated and observed data, RMSE was used. The D_index_ is a metric that describes how well observed and simulated data match, and it varies between one (complete agreement) to zero (no agreement). When the D_index_ close to 1 and a low RMSE value indicate strong agreement between observed and simulated results (Kadiyala et al., 2015; Baishya, Mishra & Sengupta, 2024).

\begin{eqnarray*}PD= \frac{100\times \left( {S}_{i}-{O}_{i} \right) }{{O}_{i}} \end{eqnarray*}

\begin{eqnarray*}RMSE={ \left[ \frac{\sum _{i=1}^{n}{ \left( {S}_{i}-{O}_{i} \right) }^{2}}{n} \right] }^{0.5} \end{eqnarray*}

\begin{eqnarray*}{r}^{2}= \frac{{ \left[ \sum _{i=1}^{n} \left( {O}_{i}-\underline{O} \right) \times \left( {S}_{i}-\underline{S} \right) \right] }^{2}}{\sum _{i=1}^{n}{ \left[ {O}_{i}-\underline{O} \right] }^{2}\times \sum _{i=1}^{n}{ \left[ {S}_{i}-\underline{S} \right] }^{2}} \end{eqnarray*}

\begin{eqnarray*}{D}_{index}=1- \frac{\sum _{i=1}^{n}{ \left( {S}_{i}-{O}_{i} \right) }^{2}}{\sum _{i=1}^{n}{ \left[ \left\vert {S}_{i}-\underline{O} \right\vert + \left\vert {O}_{i}-\underline{O} \right\vert \right] }^{2}} \end{eqnarray*}

where S_i_: model simulated values, O_i_: observed values, n: number of observations, O: mean of observed values, S: mean of simulated values.

### Climate change scenarios and data analysis

To evaluate the potential impacts of climate change, three global climate models (GCMs) and two Representative Concentration Pathways scenarios (RCP4.5 and 8.5) were considered in the study. RCP4.5 describes a stabilization pathway in which radiative forcing is projected to stabilize around 4.5 w m^−2^ by 2100, whereas RCP8.5 depicts a high-emission scenario that is expected to result in the largest increases in both global temperature and radiative forcing (IPCC, 2023).

The global climate models HadGEM2-ES, MPI-ESM-MR, and GFDL-ESM2M were selected to generate climate projections. The related datasets, representing two emission scenarios for each model, were provided by the Turkish State Meteorological Service (TSMS), which applied dynamic downscaling with the RegCM4.3.4 regional climate model at a spatial resolution of 20 km. To reduce systematic deviations, bias correction was applied by comparing the model outputs for the reference period (1970–2000) with observed meteorological station data. These corrections, performed on a daily basis for each variable and model dataset, improved the alignment of climate inputs with local conditions and enhanced the robustness of subsequent crop model simulations. This work focuses on evaluating the impacts of climate change for three time periods: near-future (2016–2040), mid-future (2041–2070), and far-future (2071–2098). The impacts of climatic changes were investigated by comparing these periods with the reference years (1970–2000) mentioned above.

Atmospheric CO_2_ concentrations in the study were assigned according to the Representative Concentration Pathways scenarios (RCP 4.5 and RCP 8.5). Period means were calculated from the official RCP concentration series and are consistent with the updated greenhouse gas concentration pathways reported by Meinshausen et al. (2020). Under the RCP 4.5 scenario, the representative CO_2_ levels were approximately 436 ppm for the near-future (2016–2040), 509 ppm for the mid-future (2041–2070), and 534 ppm for the far-future (2071–2098). Under the RCP 8.5 scenario, the corresponding averages were about 450 ppm, 593 ppm, and 846 ppm, respectively. For the reference period 1970–2000, a representative atmospheric CO_2_ concentration of ∼347 ppm was used, consistent with observed global means (NOAA Global Monitoring Laboratory, 2023). For each simulation, the DSSAT model was operated by modifying and entering the corresponding CO_2_ level as an input parameter, based on the values described above.

## Results

### Calibration results of the DSSAT-CERES-rice model

Table 1 presents the cultivar genetic coefficients used in the model, including the default values and those calibrated with the GLUE method, along with their descriptions for rice. In the calibration process of the DSSAT model, it is known that some stress factors such as water deficit and nutrient stress can cause poor estimation of the coefficients (Jones et al., 2003; Hoogenboom et al., 2010; Hoogenboom et al., 2019). Some of the Ceres sub-models of the DSSAT (such as CERES-Wheat, CERES-Maize, *etc*.) have limitations in their ability to accurately simulate the effects of water. This is due to the model’s emphasis on meteorological variables, with limited consideration given to water and nitrogen effects (Zhou, Liu & Chen, 2025). Calibration results of phenological stages under different irrigation coefficients for rice are presented in Table 2.

**Table 1:** Default and calibrated cultivar genetic coefficients of rice.

Rice genetic coefficient	Description of the coefficient	Default coefficient values	Calibrated coefficient values
P1	Time period (expressed as growing degree days (GDD) in deg.C above a base temperature of 9 °C) from seedling emergence during which the rice plant is not responsive to changes in photoperiod. Range: 150–800	Flexible	385.9
P2O	Critical photoperiod or the longest day length (in hours) at which the development occurs at a maximum rate. Range 11–13 h	12 h	10.90
P2R	The extent to which phasic development leading to panicle initiation is delayed (expressed as GDD in deg.C) for each hour increase in photoperiod above P2O. 5–300 deg.C	Lower range	100.1
P5	Time period in GDD deg.C) from beginning of grain filling (3 to 4 days after flowering) to physiological maturity with a base temperature of 9 °C. Range: 150–850 deg.C	Flexible depending on P1, P2O, P2R	320.0
G1	Potential spikelet number coefficient as estimated from the number of spikelets per g of main culm dry weight (less leaf blades and sheaths plus spikes) at anthesis. Range: 50–75 ng^−1^	55 ng^−1^	67.3
G2	Single grain weight (g) under ideal growing conditions, *i.e.*, non-limiting light, water, nutrients, and absence of pests and diseases. Range: 0.015–0.030 g	0.025 g	0.029
G3	Tillering coefficient (scalar value) relative to IR64 cultivar in the model under ideal conditions. Range: 0.7–1.3	Greater than 1.0	1.00
PHINT	Phyllochron Interval (deg.C). Time interval in degree-days for each leaf-tip to appear under non-stressed conditions. Range: 55–90 deg.C	83 deg.C	64.9
THOT	Temperature (°C) above which spikelet sterility is affected by high temperature. 25–34 °C	28 °C	28.7
TCLDP	Temperature (°C) below which panicle initiation is further delayed (other than P1, P2O and P2R) by low temperature. Range: 12–18 °C	15 °C	15.0
TCLDF	Temperature (°C) below which spikelet sterility is affected by low temperature. Range: 10–20 °C	15 °C	15.0

**Table 2:** Calibration results for the rice with varying irrigation coefficients.

Phenological Stages	I_1.00_			I_1.25_			I_1.50_		
	Simulated	Observed	Error (%)	Simulated	Observed	Error (%)	Simulated	Observed	Error (%)
Anthesis date (ADAT)	5,00	7,00	−28,57	5,00	7,00	−28,57	5,00	7,00	−28,57
Physiological maturity date (MDAT)	85,00	82,00	3,66	85,00	83,00	2,41	85,00	84,00	1,19
Emergence date (EDAT)	110,00	113,00	−2,65	110,00	113,00	−2,65	110,00	113,00	−2,65
Panicle initiation date (IDAT)	57,00	57,00	0,00	57,00	57,00	0,00	57,00	57,00	0,00

The error percentage for anthesis date (ADAT), physiological maturity date (MDAT), emergence date (EDAT) and panicle initiation date (IDAT) between simulated and observed phenological dates were varied between −28.57% and 3.66% under different irrigation coefficients. However, the DSSAT simulated the same day of the years (DOY) for both phenological stages of the rice, and all of them were in acceptable range with varying irrigation coefficients. Error statistics showed good agreement between simulated and observed values for the selected parameters. A percentage error of less than 10% between observed and predicted values suggests effective model performance (Vysakh, Ajithkumar & Rao, 2016). The results indicated that the DSSAT CERES-Rice model can estimate anthesis date, physiological maturity date, emergence date and panicle initiation date with acceptable accuracy. The agreement between observed and simulated values indicated a satisfactory performance of the model during both the calibration and validation phases (Harithalekshmi et al., 2024).

### Validation results of the DSSAT-CERES-rice model

Validation process was performed using crop growth parameters (yield, LAI, and biomass) and cultivar genetic coefficients obtained from the calibration stage. Acceptance threshold ranges of statistical analyses are presented in Table 3. Results for LAI (m^2^ m^−2^), biomass (kg ha^−1^) and yield (kg ha^−1^) parameters under three irrigation coefficients for the validation process of the DSSAT-CERES-Rice model are presented in Table 4.

**Table 3:** Acceptance threshold ranges of statistical analyses used in the validation and the verification stages of the model.

Statistical analysis	Excellent	Good	Satisfactory	Unsatisfactory		
nRMSE	nRMSE<10%	10% < nRMSE < 20%	20% < nRMSE < 30%	nRMSE > 30%		
Dindex	Very good	Good	Moderate good	Moderately poor	Poor	Very poor
	d≥0.9	0.80 < d < 0.89	0.65 < d < 0.79	0.50 < d < 0.64	0.25 < d < 0.49	d<0.25

**Notes.**

Recommendations by follows; D_index_, Akumaga, Tarhule & Yusuf (2017); nRMSE, Jamieson, Porter & Wilson (1991); Sing, Knapp & Demissie (2004).

**Table 4:** Validation results for the rice with varying irrigation coefficients.

Irrigation levels	LAI (m^2^ m^−2^)		Biomass (kg ha^−1^)		Yield (kg ha^−1^)	
	Obs	Sim	Obs	Sim	Obs	Sim
I_1.00_	3.41	2.88	5,214	5,116	2,188	2,247
	4.22	3.42	7,115	7,203	4,117	4,226
	4.10	3.45	8,426	8,566	5,487	5,531
	5.76	4.53	11,544	11,661	6,188	6,257
	5.25	4.91	12,755	12,914	6,618	6,834
	5.32	4.32	15,873	14,508	6,763	7,091
*RMSE*	0.81		568.50		296.90	
*nRMSE*	17.40		5.60		5.60	
*D*_*index*_	0.79		0.99		0.99	
*R* ^2^	0.87		0.98		0.99	
I_1.25_	3.49	3.41	5,597	5,644	2,412	2,367
	4.28	3.98	7,812	7,869	5,412	4,469
	4.40	4.00	9,296	9,336	5,941	5,855
	6.21	4.94	12,603	12,642	6,633	6,545
	5.50	4.05	13,822	13,936	7,290	7,131
	6.00	4.85	18,080	15,467	7,563	7,392
*RMSE*	0.94		1,068.44		693.16	
*nRMSE*	18.86		9.54		9.54	
*D*_*index*_	0.73		0.98		0.99	
*R* ^2^	0.86		0.96		0.96	
I_1.50_	3.70	3.30	5,611	5,542	2,280	2,339
	4.85	3.86	7,847	7,736	4,523	4,412
	4.50	3.88	9,256	9,175	5,906	5,778
	6.48	4.87	13,154	12,415	6,527	6,478
	5.79	4.02	13,880	13,702	7,110	7,061
	6.32	3.82	20,500	15,246	8,057	7,320
*RMSE*	1.50		2,168.18		538.59	
*nRMSE*	28.44		18.52		9.39	
*D*_*index*_	5.59		0.28		0.99	
*R* ^2^	0.57		0.91		0.98	

**Notes.**

Obs, Observed; Sim, Simulated.

**Table 5:** Results of bias correction for climatic parameters compared to reference period.

CGMs	Periods	Tmax (°C)		Tmin (°C)		Precipitation (mm)	
		RCP 4.5	RCP 8.5	RCP 4.5	RCP 8.5	RCP 4.5	RCP 8.5
GFDL-ESM2M	2016–2040	6.92	7.09	4.51	4.75	1.62	0.68
	2041–2070	7.28	8.21	4.92	5.68	1	0.56
	2071–2098	7.47	9.68	5.07	6.95	0.62	0.08
HadGEM2-ES	2016–2040	5.51	5.81	3.94	4.31	−0.25	−0.22
	2041–2070	6.24	6.99	4.54	5.33	−0.56	−0.5
	2071–2098	6.83	8.73	5.16	7.08	−0.29	−0.24
MPI-ESM-MR	2016–2040	4.38	4.35	2.93	2.95	0.34	0.29
	2041–2070	4.66	5.57	3.25	3.91	0.38	0.32
	2071–2098	5.16	7.65	3.61	5.64	0.34	0.03

**Figure 2:**
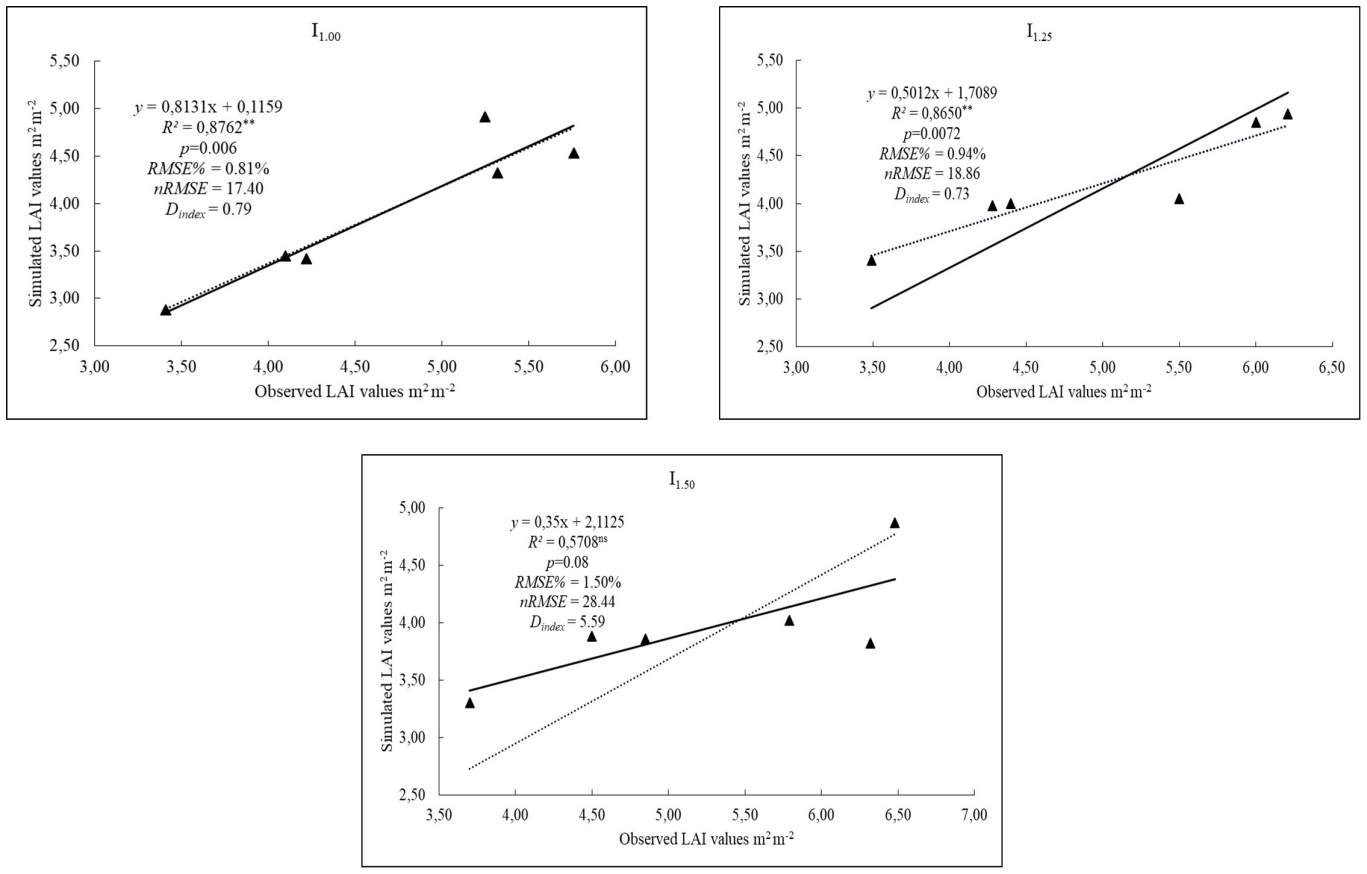
The results of the observed and simulated LAI (m^2^ m^−2^) values of rice for I_1.00_, I_1.25_, I_1.50_ irrigation levels.

Figure 2 presents the results for LAI of rice under different irrigation coefficients for the validation process. For LAI, mean observed values ranged from 4.68 to 5.27 across the irrigation coefficients, while the corresponding simulated values were 3.92, 4.21, and 3.96, respectively. Validation results of the DSSAT-CERES-Rice model showed that nRMSE between the simulated and observed LAI values were 17.40%, 18.86% and 28.44% for the different irrigation coefficients (I_1.00_, I_1.25_ and I_1.50_) respectively. nRMSE values fit well for I_1.00_ and I_1.25_ with a rating of good, while I_1.50_ irrigation level was satisfactory. The D_index_ values between observed and simulated values for LAI were 0.79, 0.73 and 0.59 respectively. D_index_ values for I_1.00_ and I_1.25_ showed moderately good agreement while I_1.50_ was poor. Zhou, Liu & Chen (2025) reported that, under water-deficit conditions, the nRMSE values between simulated and observed LAI of millet ranged from 8.27% to 18.35%. They also stated that the DSSAT-CERES-Millet model tends to underestimate LAI values when simulating different irrigation treatments.

**Figure 3:**
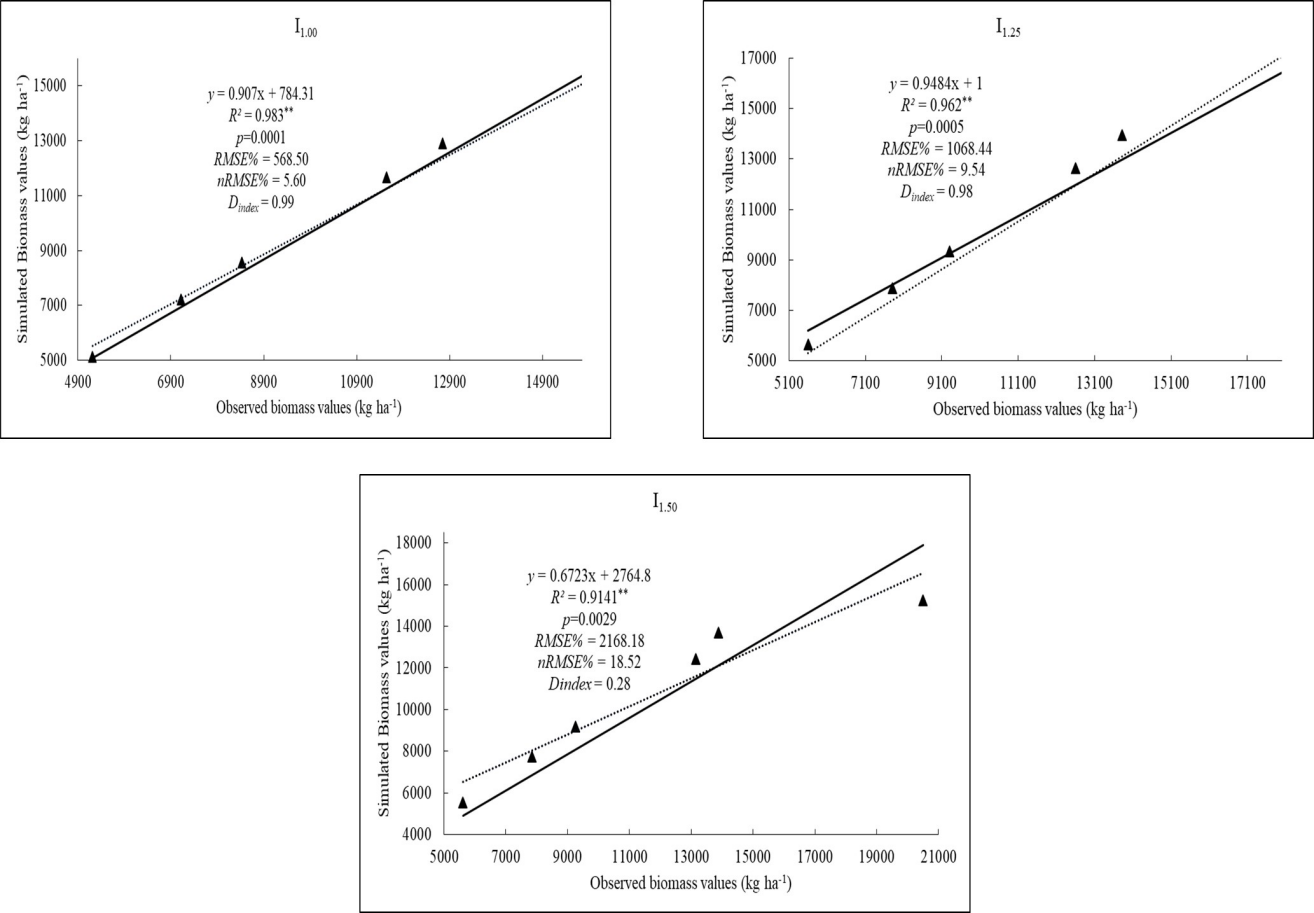
The results of the observed and simulated biomass (kg ha^−1^) values of rice for I_1.00_, I_1.25_, I_1.50_ irrigation levels.

The results indicate that biomass simulated by the model ranged from 5.116 to 15.467 kg ha^−^^1^ under different irrigation coefficients, while the observed biomass values ranged from 5.214 to 20.500 kg ha^−^^1^. Figure 3 shows that the simulated biomass values with different irrigation coefficients were closely related to the observed data with nRMSE values of 5.60%, 9.54%, and 18.52% respectively. Irrigation levels I1.00 and I1.25 were rated as excellent, demonstrating the ability of the calibrated DSSAT-CERES-Rice model to accurately simulate rice biomass. Dindex statistics showed very good agreement between simulated and observed biomass values for I_1.00_ and I_1.25_ (0.99–0.98). The I_1.50_ level showed poor agreement with a Dindex value of 0.28.

**Figure 4:**
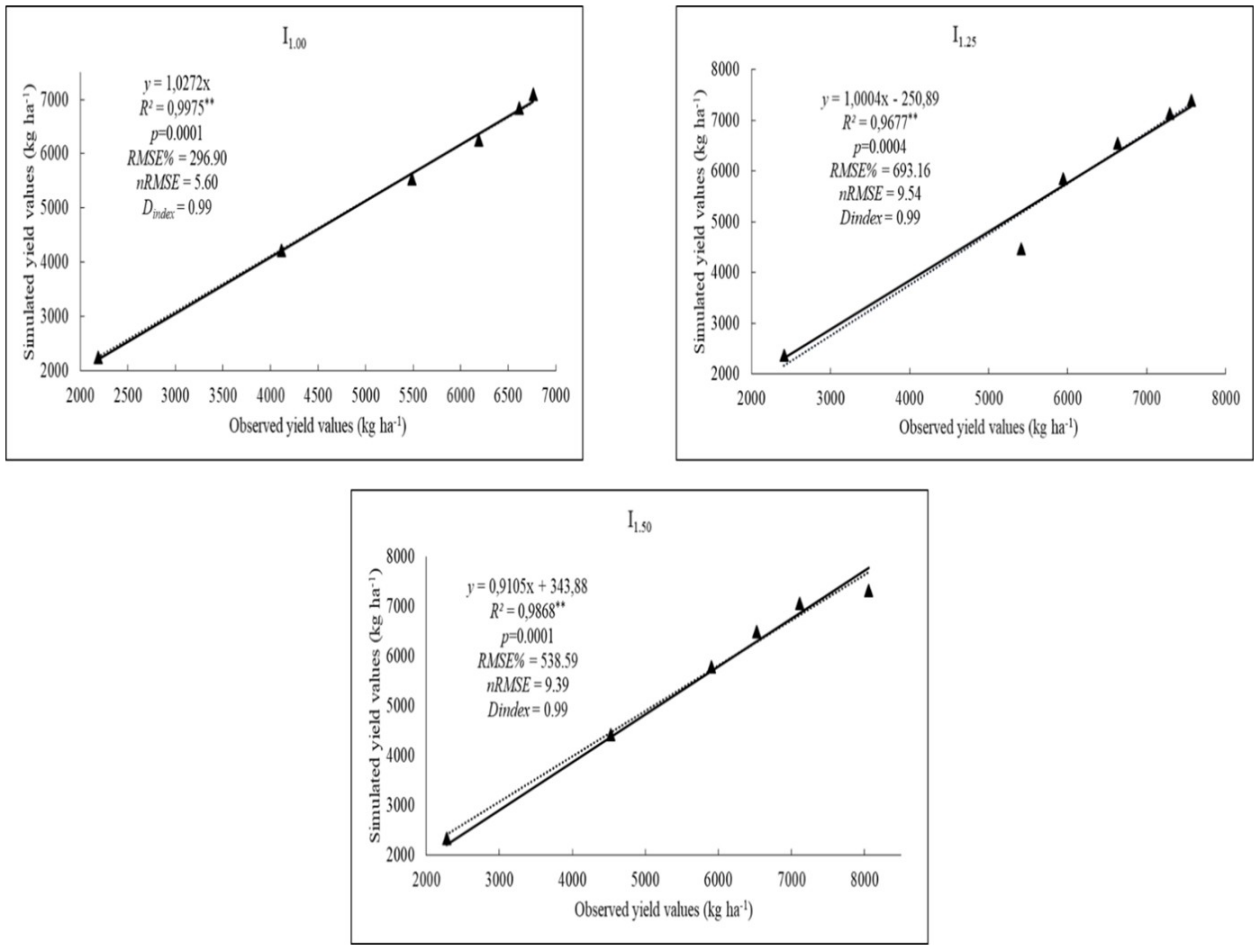
The results of the observed and simulated yield (kg ha^−1^) values of rice for I_1.00_, I_1.25_, I_1.50_ irrigation levels.

Figure 4 shows the results of the validation of the DSSAT-CERES-Rice model considering to yield values. The observed and simulated yield of rice under different irrigation levels was excellent (nRMSE < 10%) (Fig. 4). Results indicate that the simulated yield parameter strongly agreed with observed values. nRMSE values were obtained as 5.60%, 9.54%, and 9.39%, respectively. D_index_ values were ranked as very good with the same values of 0.99 in all levels (I_1.00_, I_1.25_ and I_1.50_). There are many studies available that confirm the DSSAT-CERES-Rice model can estimate yield values with high accuracy and consistent with this study. According to Zhang, Zhou & He (2025), the RMSE between simulated and observed yield was 7.8%, and the d-index was 0.97, indicating strong model performance. Similarly, Mandapati et al. (2024) reported a correlation coefficient of 0.81 and a d-index of 0.83 for rice yield predictions, further supporting the reliability of the model.

### Assessment of climate projection data

Table 5 presents the bias-correction results of the three global climate models (GFDL-ESM2M, HadGEM2-ES, MPI-ESM-MR) with two RCP scenarios (RCP 4.5 and 8.5), compared to the reference years (1970–2000).

According to Table 5, maximum temperatures were expected to rise steadily in all models and scenarios. In GFDL-ESM2M, the increase ranged from 6.9–7.1 °C in 2016–2040 to 7.5–9.7 °C in 2071–2098. The HadGEM2-ES model indicated a rise from 5.5–5.8 °C to 6.8–8.7 °C, while MPI-ESM-MR showed comparatively smaller changes, from 4.4 °C in the near future to 5.2–7.7 °C in the late future. Minimum temperature values also increased in all models. In the GFDL-ESM2M model, Tmin increased from 4.5–4.8 °C during 2016–2040 to 5.1–7.0 °C in 2071-2098. HadGEM2-ES showed an increase from 3.9–4.3 °C to 5.2–7.1 °C, whereas MPI-ESM-MR exhibited a more moderate rise, from 2.9–3.0 °C in the early projection period to 3.6–5.6 °C toward the end of the future. Precipitation showed a decrease compared with the reference period. In the GFDL-ESM2M model, values decreased from 1.62–0.68 mm in 2016–2040 to 0.62–0.08 mm in 2071–2098. The HadGEM2-ES model showed consistently negative anomalies, ranging from −0.25 to −0.56 mm, whereas the MPI-ESM-MR model indicated only slight reductions, decreasing from 0.29–0.38 mm in the near-term period to 0.34–0.03 mm toward the end of the future.

These projections clearly showed that RCP 8.5 resulted in greater temperature increases than RCP 4.5 across all time periods, with the GFDL-ESM2M model producing the highest values and the MPI-ESM-MR model the lowest. For precipitation, the models exhibited different patterns, with the largest decreases occurring under the GFDL-ESM2M and HadGEM2-ES models, while the MPI-ESM-MR model showed only slight reductions.

**Figure 5:**
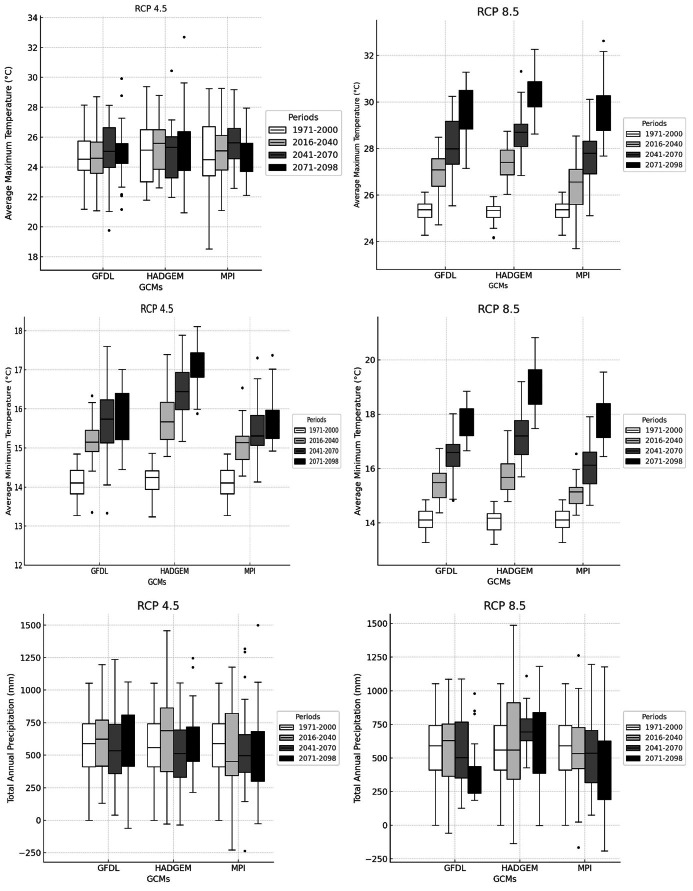
Climate change projections results.

### Climate change projections for future time periods

The climate projection results for maximum temperature (Tmax), minimum temperature (Tmin), and precipitation are presented in Fig. 5. The figure summarizes the projected changes relative to the reference period (1971–2000) for three future periods defined as the near future (2016–2040), mid-future (2041–2070), and late-future (2071–2098) under both RCP 4.5 and RCP 8.5 scenarios. Recent regional assessments in Türkiye, such as the study by Demircan et al. (2017) have highlighted that projections based on HadGEM2-ES, MPI-ESM-MR, and GFDL-ESM2M consistently indicate rising temperatures and decreasing precipitation throughout the 21st century, which aligns with the patterns identified in the present results.

Changes in maximum temperature indicated a persistent warming signal throughout the century, with the intensity varying by scenario, model, and period. Under RCP 4.5, the increase remained moderate, reaching its highest levels toward the end of the century. In contrast, RCP 8.5 produced stronger increases, particularly after the mid-century period. Among the models, HadGEM2-ES tended to show the greatest rise in Tmax, especially during 2071–2098, whereas MPI-ESM-MR presented comparatively smaller increases. GFDL-ESM2M generally showed intermediate values, suggesting a level of warming between the two. Similar patterns have been reported in regional assessments, where RCP 8.5 consistently projected greater warming than RCP 4.5 (Öztürk et al., 2015).

### Impacts of climate change on the growth stages of rice

**Figure 6:**
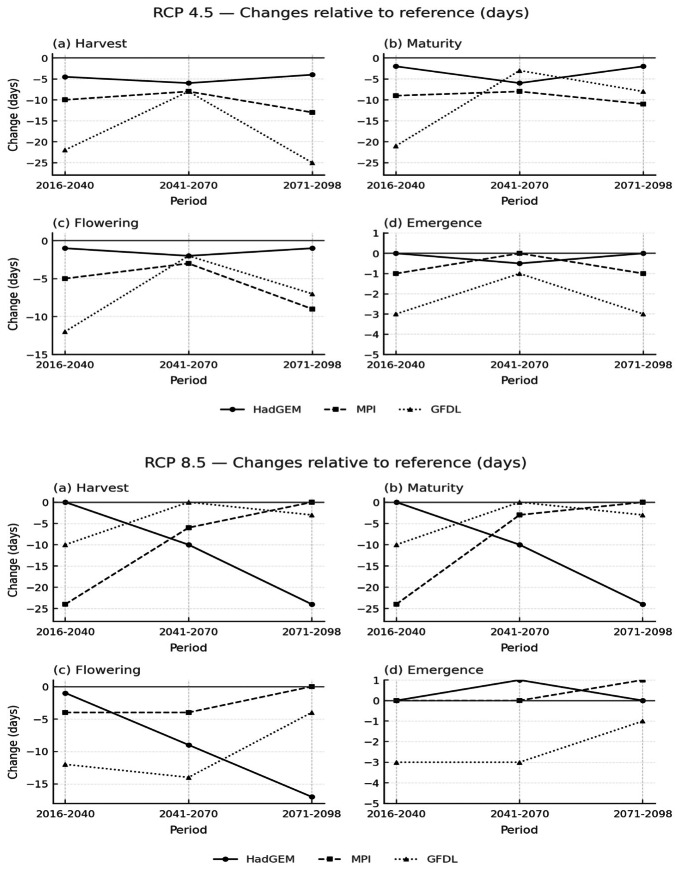
Climate change effects on rice growth stages.

In this section of the study, the impacts of climate change on the growth stages of rice were evaluated under two Representative Concentration Pathways (RCP 4.5 and RCP 8.5). Three regional climate models: HadGEM2-ES, GFDL-ESM2M, and MPI-ESM-MR were considered to assess future changes, as shown in Fig. 6. The analysis was conducted for three separate time periods: near future (2016–2040), mid-future (2041–2070), and late-future (2071–2098). The aim was to investigate how flowering, maturity, and harvest dates are expected to shift under different emission scenarios and climate model projections compared with the reference period.

 Under the RCP 4.5 scenario, the flowering stage is expected to occur about 3–5 days earlier than the reference period, especially during the mid and late-future periods (2041–2070 and 2071–2098). For instance, in MPI-ESM-MR the flowering date showed a decrease of nearly 4 days, while in GFDL the advancement reached about 12–14 days depending on the period. Likewise, maturity and harvest dates showed a progressive advancement of roughly 5–10 days in the same periods, pointing to a gradual decrease in the length of the growth cycle.

By comparison, the RCP 8.5 scenario indicated stronger changes. Flowering is expected to occur over 15 days earlier in the late-future period (2071–2098), particularly in HadGEM where the decrease reached −17 days. Harvest timing also shifted markedly, with decreases of nearly 20–24 days in HadGEM by the late-future period. Guo et al. (2019a), Guo et al. (2019b) also emphasized that multi-model assessments consistently revealed earlier flowering and shortened growth duration of rice under high-emission scenarios. Among the climate models, HadGEM2-ES consistently showed the largest decrease in both maturity and harvest times under RCP 8.5, while MPI-ESM-MR and GFDL-ESM2M showed more moderate decreases.

When the two scenarios are compared, the greatest decrease in harvest timing is observed in HadGEM2-ES under RCP 8.5, exceeding 20 days in the late-future (2071–2098). For maturity, the strongest decrease likewise appeared in HadGEM2-ES under RCP 8.5, whereas under RCP 4.5 the decreases remain within 10 days in all models. These findings suggest that moderate emissions (RCP 4.5) are associated with limited decreases in phenological stages, while high emissions (RCP 8.5) lead to substantial decreases, particularly evident in the maturity and harvest periods. Luo et al. (2022) confirmed that late-future projections under RCP 8.5 consistently show the strongest contractions in rice growth stages.

### Impacts of climate change on yield of the rice

**Figure 7:**
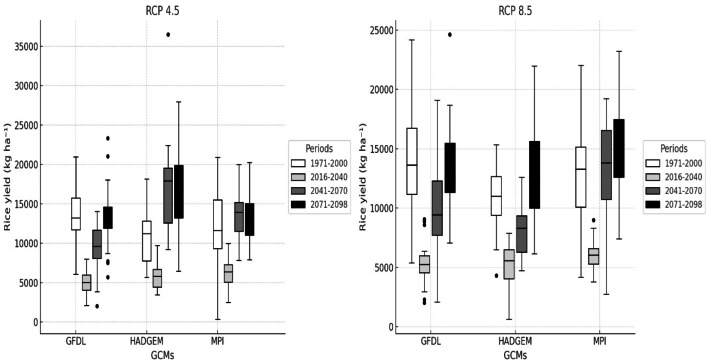
Climate change effects on rice yield for the future on irrigated conditions.

**Figure 8:**
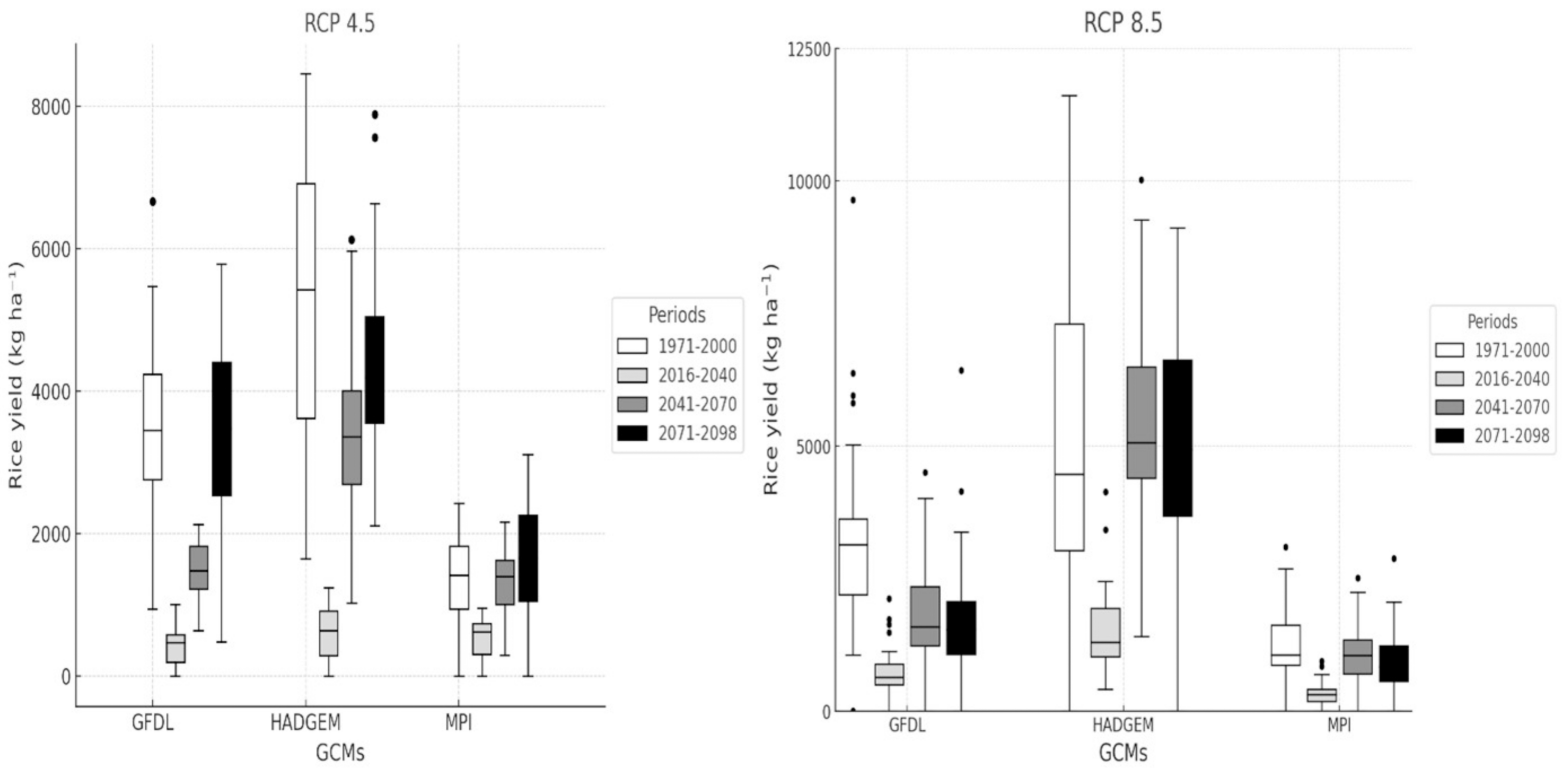
Climate change effects on rice yield for the future on not irrigated (rainfed) conditions.

Figures 7 and 8 illustrate the impacts of climate change between the projected periods. The DSSAT crop simulation model indicated that rice yields varied depending on irrigation management, climate scenario, and climate model. Under irrigated conditions, yields generally remained higher than the reference period. In the HadGEM2-ES model under RCP 8.5, the average yield in the late-future (2071–2098) reached about 7,800–8,000 kg ha^−1^, an increase of nearly 10% compared to the reference period value of 7,100 kg ha^−1^. This increase is consistent with the precipitation projections from the HadGEM2-ES climate scenarios, which partly balanced the negative effects of higher temperatures. Li et al. (2024) found that rice yields showed an increase under scenarios with higher precipitation, despite warming conditions. In contrast irrigated yields simulated by GFDL-ESM2M and MPI-ESM-MR under RCP 8.5 were close to the reference period or slightly lower, generally between 6,900 and 7,200 kg ha^−1^ reflecting the combined effects of strong warming and reduced precipitation in these models. Tran, Tseng & Chen (2025) indicated that rice yields showed a decrease under warming conditions in the absence of adaptation practices. Under RCP 4.5, irrigated yields stayed closer to the reference period, with HadGEM2-ES showing a modest increase while GFDL-ESM2M and MPI-ESM-MR remained nearly stable.

Under rainfed conditions, yields consistently showed a decrease, and this reduction became stronger in warmer scenarios. In the late-future period under RCP 8.5, HadGEM2-ES projected yields around 5,700–6,000 kg ha^−1^, a decrease of 15–20% relative to the reference period. Wang et al. (2024) reported that rice yields showed a decrease in regions where precipitation was reduced under future scenarios. GFDL-ESM2M simulated values below 5,500 kg ha^−1^, a reduction exceeding 20%, while MPI-ESM-MR projected yields between 6,000 and 6,300 kg ha^−1^, a decrease of about 10–15%. Guo et al. (2019a) and Guo et al. (2019b) found that RCP 8.5 projections frequently resulted in markedly stronger decreases in rice yield compared to more moderate scenarios. However, under RCP 4.5 the reductions were less severe, generally around 8–9% lower than the reference period, whereas under RCP 8.5 decreases reached over 30%.

## Discussion

The model’s limited ability to accurately predict phenological stages may depend on the combined effects of site-specific climate variability and cultivar-specific responses to water stress. This outcome may result from the inadequate representation of water stress in the model, which has a considerable impact on the performance of the GLUE algorithm in determining calibrated parameter sets (Yan, Jin & Wu, 2020). Although manual adjustment of soil parameters in the model’s soil module under water-stressed conditions may lead to improved accuracy, it may reduce the model’s performance under non-stressed conditions (Yao et al., 2025). In this study irrigation coefficients were selected in calibration and validation process of the model. The results showed that irrigation management can also be used as a method for model evaluation, as confirmed by statistical indicators such as RMSE, error percentage, D_index_ and R^2^. Under conditions of limited water and nitrogen availability, crops activate complex adaptive mechanisms to cope with resource constraints. These mechanisms involve a range of physiological and morphological changes, including altered root architecture, regulated stomatal behavior, and reprogrammed metabolic pathways which challenge the DSSAT-CERES model in accurately simulating crop responses (Pandey, Vengavasi & Hawkesford, 2021; Yang et al., 2021; Jones et al., 2003).

The DSSAT crop simulation model revealed that the future pattern of rice production in the Mediterranean region will be determined by greenhouse gas scenarios, climate model projections, and water availability. Under irrigated conditions, yields were generally maintained and, in some cases, slightly improved, whereas under rainfed conditions, yields decreased in all models, with stronger reductions under the high-emission scenario. When strong warming was combined with reduced precipitation, the beneficial effect of elevated CO_2_ was not sufficient to maintain yields at reference levels, and in some cases yields still decreased even under irrigated conditions. Shortened phenological duration represents another mechanism behind the yield decrease in rainfed systems. Rising temperatures accelerated flowering, maturity, and harvest, which may limit biomass accumulation and grain filling. This decrease can also be explained by increased evaporative demand and soil-moisture stress, while heat stress during reproductive stages may lower grain formation and final yield.

### Study limitations and future directions

Although simulated and observed values showed good agreement in the model, LAI values especially in I_1.50_ level showed poor agreement with 0.59 value of D_index_, low R^2^ (0.57) and satisfactory with nRMSE (28.44). This indicates that varying irrigation levels may constrain the model’s accuracy in estimating LAI. However, the GLUE cultivar coefficient calculator module of the DSSAT estimates LAI values, users should adjust coefficients with manually if needed. A similar situation was determined in simulated biomass values. However, R^2^ was high (0.91) in the I_1.50_ level, nRMSE ranked good (18.52%) while I_1.00_ and I_1.25_ ranked excellent. D_index_ showed poor range with value of 0.28 in the I_1.50_. This result can be explained by the fact that the treatments used in the model include different irrigation levels. It is recommended to consider non-stress factors during the calibration phase of the DSSAT model (Hoogenboom et al., 2019). The DSSAT-CERES-Rice model sometimes overestimated or underestimated the values of grain yield, especially under water stress conditions (Goswami & Dutta, 2020). Furthermore, DSSAT provides a simplified representation of crop physiology and stress responses and may underestimate the effects of extreme heat events or compound stresses on yield formation. In the irrigated scenarios, the model assumed a constant and unrestricted water supply; however, this does not account for possible limitations in water availability that may arise under future climatic and resource conditions.

Considering the inputs used in DSSAT, the data obtained under water and other stress conditions can be utilized in decision support processes for agricultural production. However, these data may contain inaccuracies. Therefore, it is necessary to improve the modules related to stress factors within the model, accounting for other environmental variables as well in the future studies. The DSSAT model includes modules that enable simulations under various stress conditions such as temperature and salinity. In this context, expanding studies on environmental and stress-related factors will enhance the model’s predictive capability. In the future, similar studies on different crops are expected to contribute to the adaptation processes of countries to climate change.

## Conclusions

The DSSAT model allows researchers to manage irrigation practices, including irrigation amounts, methods, and soil-water balance. This study aimed to evaluate the DSSAT-CERES-Rice crop simulation model (CSM) using field observations and simulated data from the model with various parameters including three different irrigation levels (I_1.00_, I_1.25_ and I_1.50_), phenological stages (anthesis date, physiological maturity date, panicle initiation date and emergence date) for calibration, and growth parameters (yield, biomass and LAI) for validation. The accuracy of the results obtained from the DSSAT model outputs can be validated through field experiments, and these findings can be shared with farmers to help them manage their agricultural production more sustainably. Furthermore, such results may serve as a guiding tool for countries in shaping their future agricultural production strategies and policies. By employing the model as a decision-support tool, farmers can also estimate yield under water-limited conditions, thus enhancing their ability to manage production potential more efficiently in the rice areas. The results revealed that comparison between observed and simulated values were within the acceptance range in both the calibration and validation processes, indicating that the DSSAT-CERES-Rice model can be used as an effective tool to simulate rice production in the study area of the Mediterranean region. From the perspective of the model’s response to climate change, irrigated conditions indicated greater resilience under changing climatic conditions, whereas the vulnerability of rainfed conditions underlines the importance of water availability as a limiting factor in future production. This situation highlights the need for more intensive research on the possible impacts of climate change on water resources in the coming years. Following accurate calibration and validation, the DSSAT crop simulation model facilitated the effective incorporation of climate change scenarios in this study.

## Supplemental Information

10.7717/peerj.20965/supp-1 Supplemental Information 1 Calibration and validation progresses of the Dssat Model Statistical analyse results of the DSSAT model for calibration and validation stages

10.7717/peerj.20965/supp-2 Supplemental Information 2 Results of crop growth parameters PlantGro.out file includes crop growth parameters such as biomass (CWAD), leaf area index (LAID) and yield (GWAD) for the day of years.

10.7717/peerj.20965/supp-3 Supplemental Information 3 Overview of the results Results of the simulation including experiment

10.7717/peerj.20965/supp-4 Supplemental Information 4 Experiment section of Xbuild Screenshot of experiment section in Xbuild file in DSSAT

10.7717/peerj.20965/supp-5 Supplemental Information 5 T file in DSSAT Created experimental T file

10.7717/peerj.20965/supp-6 Supplemental Information 6 Climate data of study area Climate data of created weather file in DSSAT

10.7717/peerj.20965/supp-7 Supplemental Information 7 Cultivar section of Xbuild Screenshot of cultivar section in Xbuild file in DSSAT

10.7717/peerj.20965/supp-8 Supplemental Information 8 Field section of Xbuild Screenshot of field section in Xbuild in DSSAT

10.7717/peerj.20965/supp-9 Supplemental Information 9 A file in DSSAT Created experimental A file

10.7717/peerj.20965/supp-10 Supplemental Information 10 Initial section of Xbild Screenshot of initials section in Xbuild file in DSSAT

10.7717/peerj.20965/supp-11 Supplemental Information 11 Cultivar coefficients Calibrated cultivar coefficients (given name YARR)

10.7717/peerj.20965/supp-12 Supplemental Information 12 Irrigation section of Xbuild Screenshot of irrigation section in Xbuild file in DSSAT

10.7717/peerj.20965/supp-13 Supplemental Information 13 Planting section of Xbuild Screenshot of planting section in Xbuild file in DSSAT

10.7717/peerj.20965/supp-14 Supplemental Information 14 Simulation options of Xbuild Screenshot of simulation options section in Xbuild file in DSSAT

10.7717/peerj.20965/supp-15 Supplemental Information 15 Soil analysis section of Xbuild Screenshot of soil analysis section in Xbuild file in DSSAT

10.7717/peerj.20965/supp-16 Supplemental Information 16 English codebook Explanation of the codes in the data

10.7717/peerj.20965/supp-17 Supplemental Information 17 DSSAT results for reference period

10.7717/peerj.20965/supp-18 Supplemental Information 18 DSSAT results for future periods

10.7717/peerj.20965/supp-19 Supplemental Information 19 GFDL Model RCP 4.5 precipitaitons results

10.7717/peerj.20965/supp-20 Supplemental Information 20 HADGEM model RCP 4.5 precipitations results

10.7717/peerj.20965/supp-21 Supplemental Information 21 MPI model RCP 4.5 precipitations results

10.7717/peerj.20965/supp-22 Supplemental Information 22 GFDL model RCP 4.5 tmax results

10.7717/peerj.20965/supp-23 Supplemental Information 23 MPI model RCP 4.5 tmax results

10.7717/peerj.20965/supp-24 Supplemental Information 24 Hadgem model RCP 4.5 tmax results

10.7717/peerj.20965/supp-25 Supplemental Information 25 GFDL model RCP 4.5 tmin results

10.7717/peerj.20965/supp-26 Supplemental Information 26 Hadgem model RCP 4.5 tmin results

10.7717/peerj.20965/supp-27 Supplemental Information 27 MPI model RCP 4.5 tmin results

10.7717/peerj.20965/supp-28 Supplemental Information 28 GFDL model RCP 8.5 precipitation results

10.7717/peerj.20965/supp-29 Supplemental Information 29 MPI model RCP 8.5 Precipitation results

10.7717/peerj.20965/supp-30 Supplemental Information 30 Hadgem model RCP 8.5 precipitation results

10.7717/peerj.20965/supp-31 Supplemental Information 31 GFDL model RCP 8.5 tmax results

10.7717/peerj.20965/supp-32 Supplemental Information 32 HADGEM model RCP 8.5 tmax results

10.7717/peerj.20965/supp-33 Supplemental Information 33 MPI model RCP 8.5 tmax results

10.7717/peerj.20965/supp-34 Supplemental Information 34 GFDL model RCP 8.5 tmin results

10.7717/peerj.20965/supp-35 Supplemental Information 35 MPI model RCP 8.5 tmin results

10.7717/peerj.20965/supp-36 Supplemental Information 36 HADGEM model RCP 8.5 tmin results

## Additional Information and Declarations

### Data Availability

The following information was supplied regarding data availability: The data is available in the Supplemental File.
