# Supplementary figures and images for: Assessing rice yield responses to climate change scenarios using a crop simulation model
Source: PeerJ. 2026 Mar 12;14:e20965. doi: 10.7717/peerj.20965 (PMC12989151; doi:10.7717/peerj.20965)

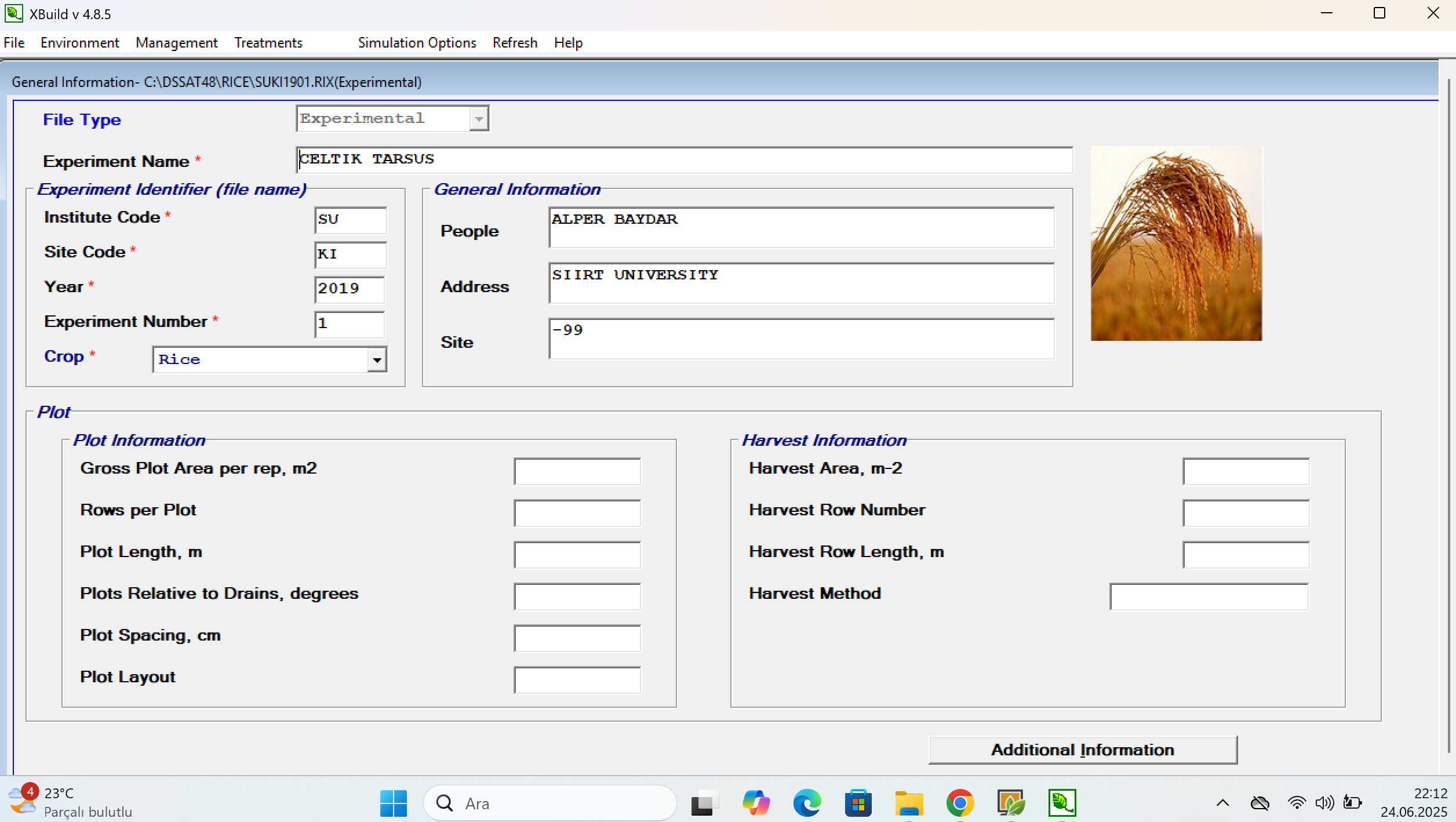

Supplement: Supplemental Information 4 — Screenshot of experiment section in Xbuild file in DSSAT [file peerj-14-20965-s004.jpg]

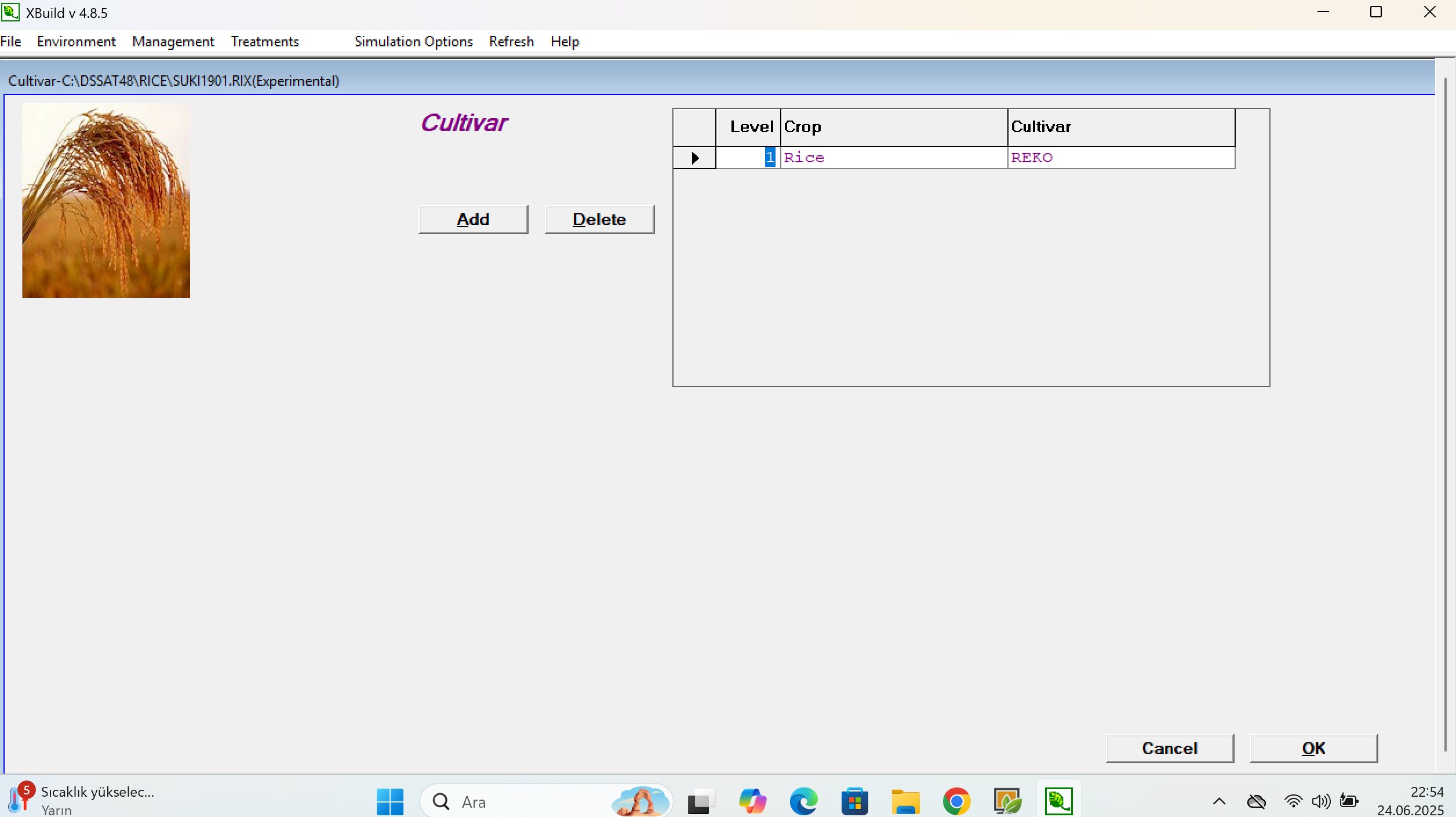

Supplement: Supplemental Information 7 — Screenshot of cultivar section in Xbuild file in DSSAT [file peerj-14-20965-s007.jpg]

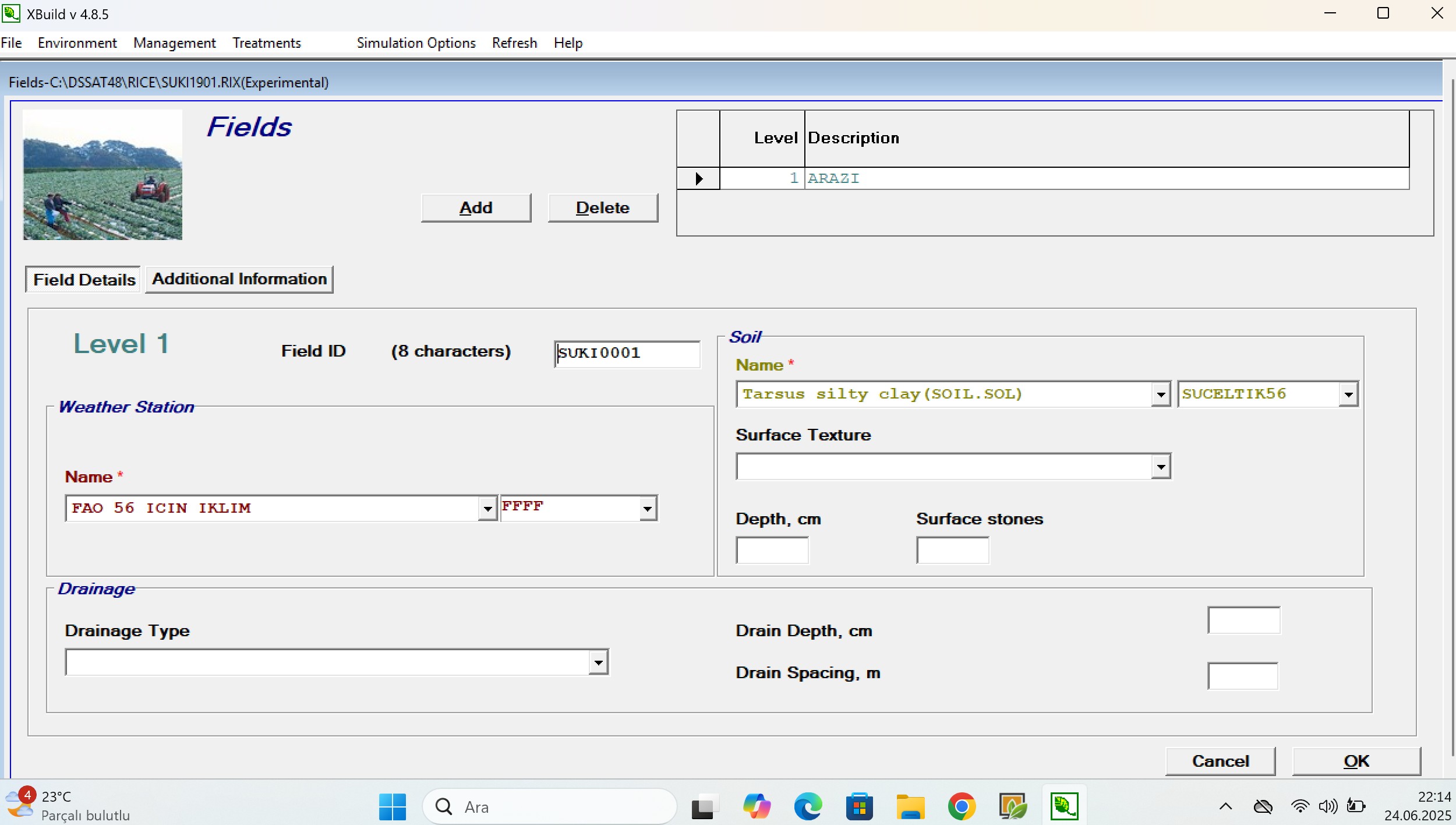

Supplement: Supplemental Information 8 — Screenshot of field section in Xbuild in DSSAT [file peerj-14-20965-s008.jpg]

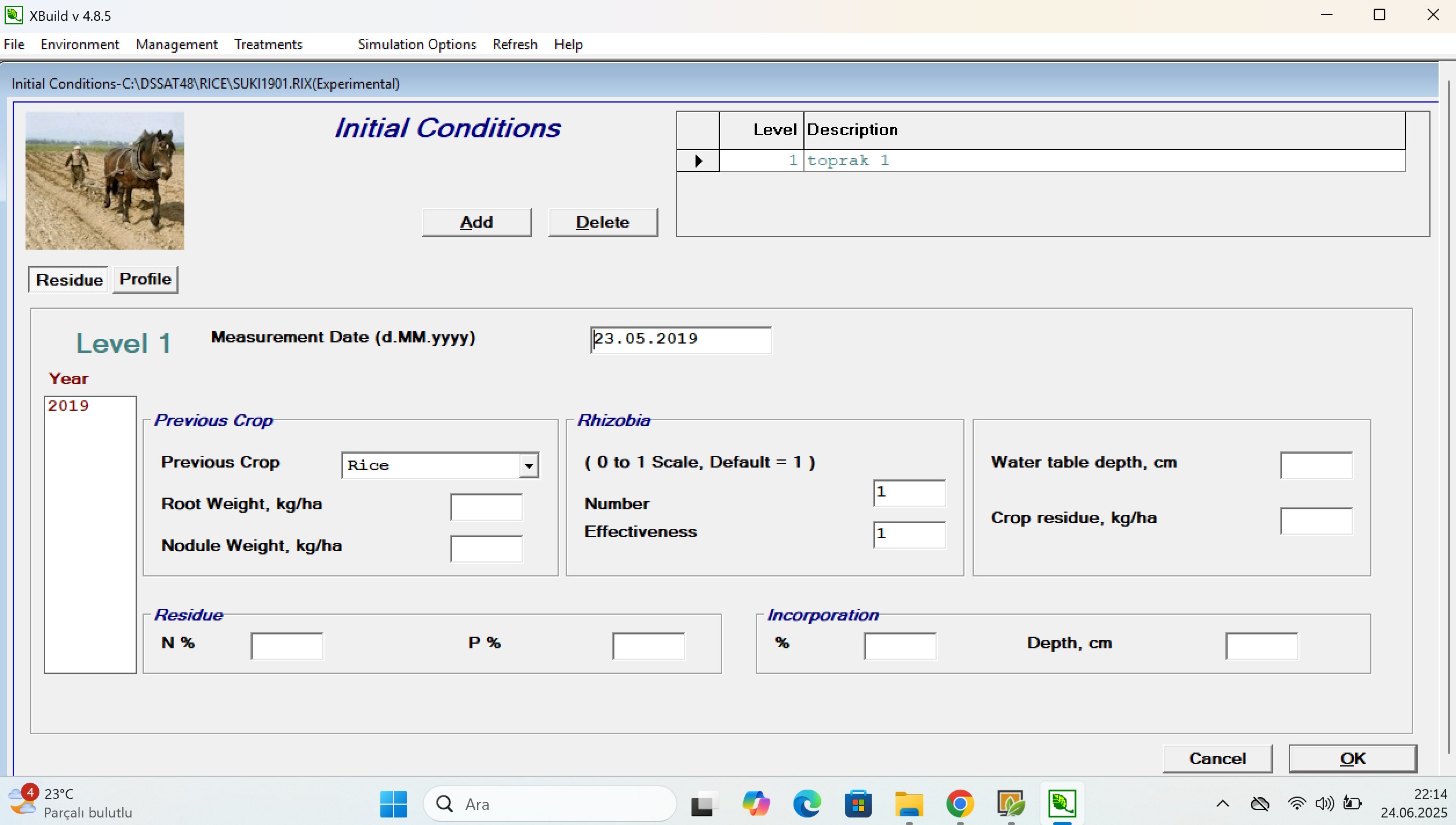

Supplement: Supplemental Information 10 — Screenshot of initials section in Xbuild file in DSSAT [file peerj-14-20965-s010.jpg]

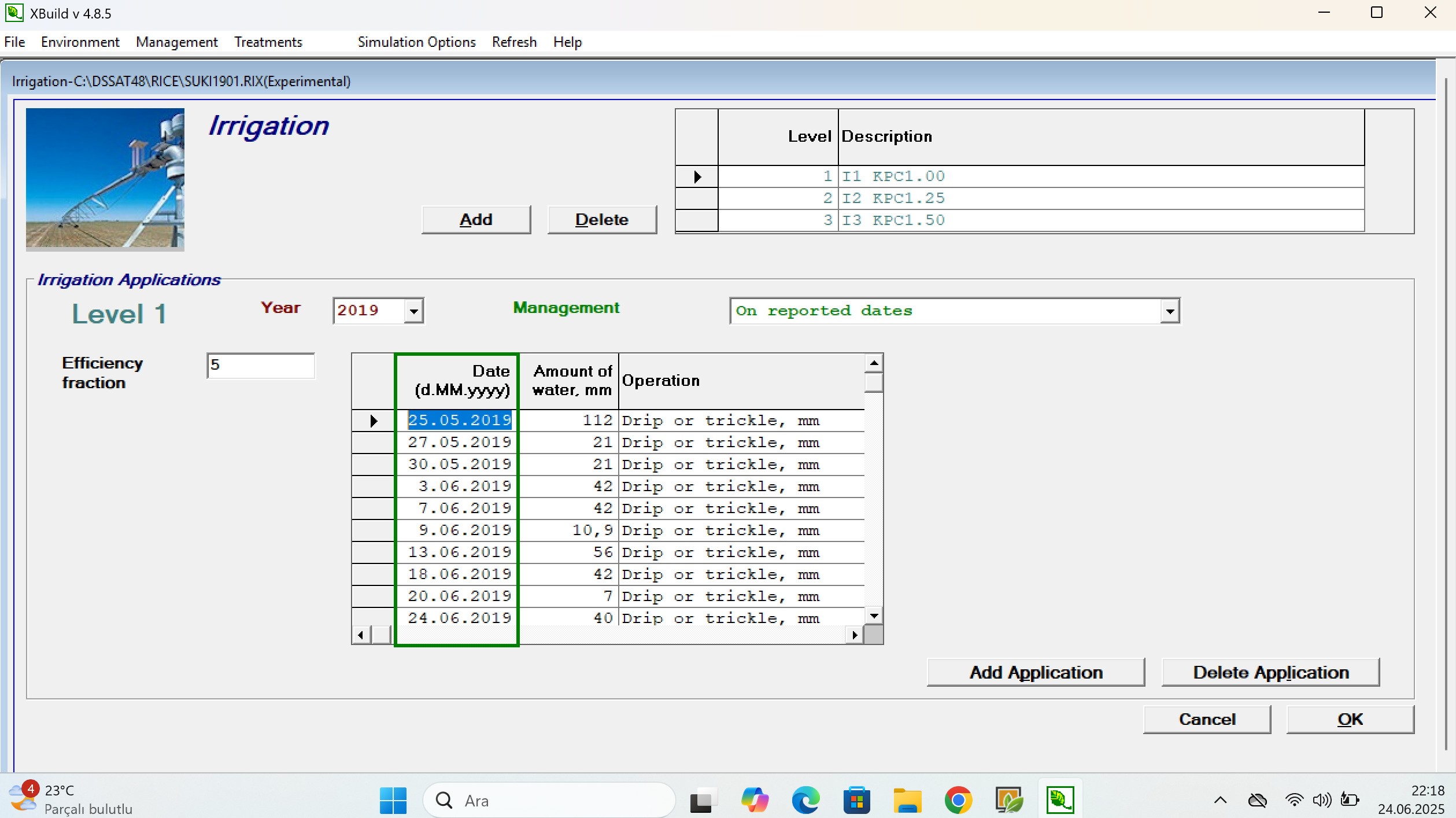

Supplement: Supplemental Information 12 — Screenshot of irrigation section in Xbuild file in DSSAT [file peerj-14-20965-s012.jpg]

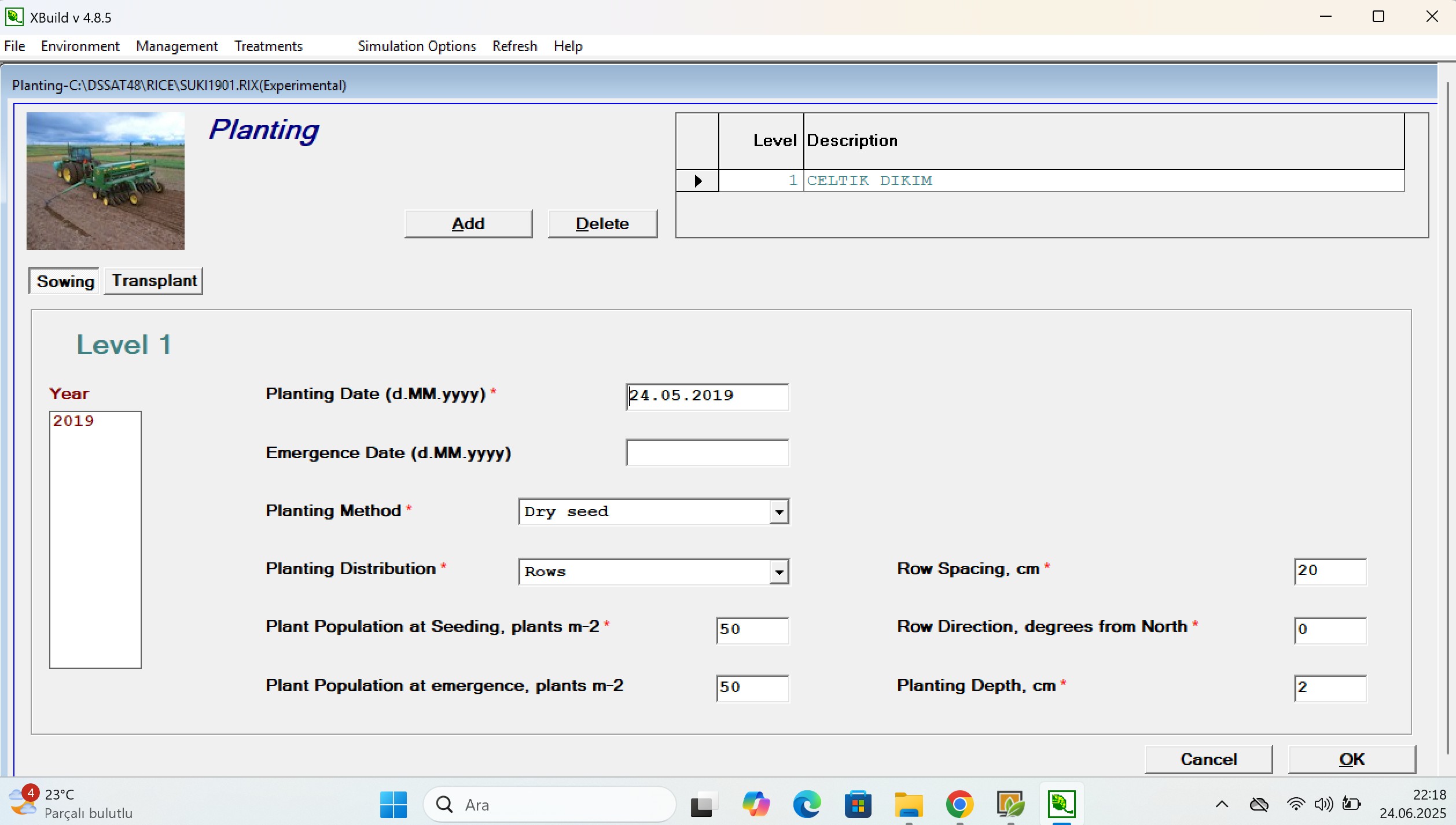

Supplement: Supplemental Information 13 — Screenshot of planting section in Xbuild file in DSSAT [file peerj-14-20965-s013.jpg]

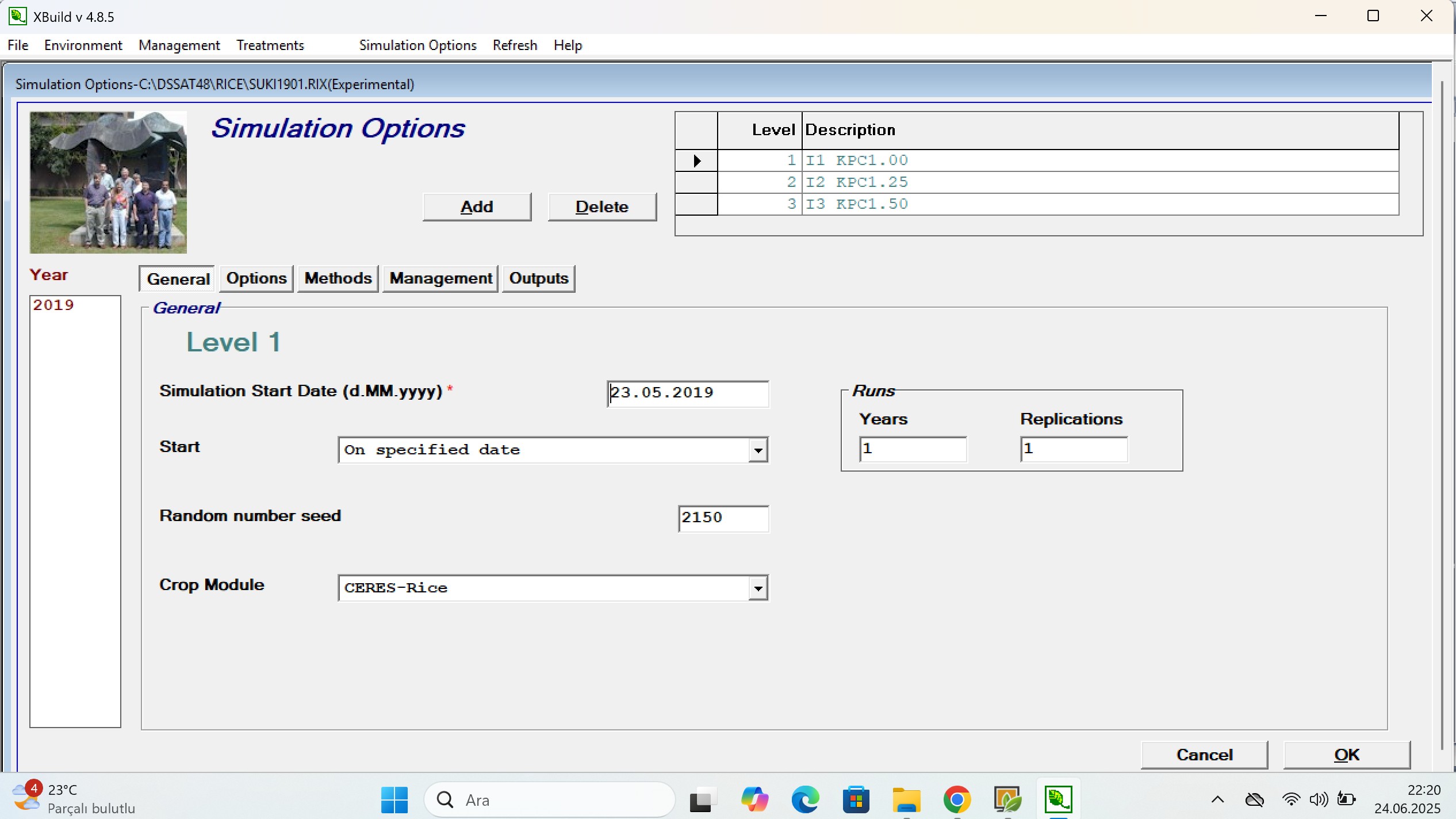

Supplement: Supplemental Information 14 — Screenshot of simulation options section in Xbuild file in DSSAT [file peerj-14-20965-s014.jpg]

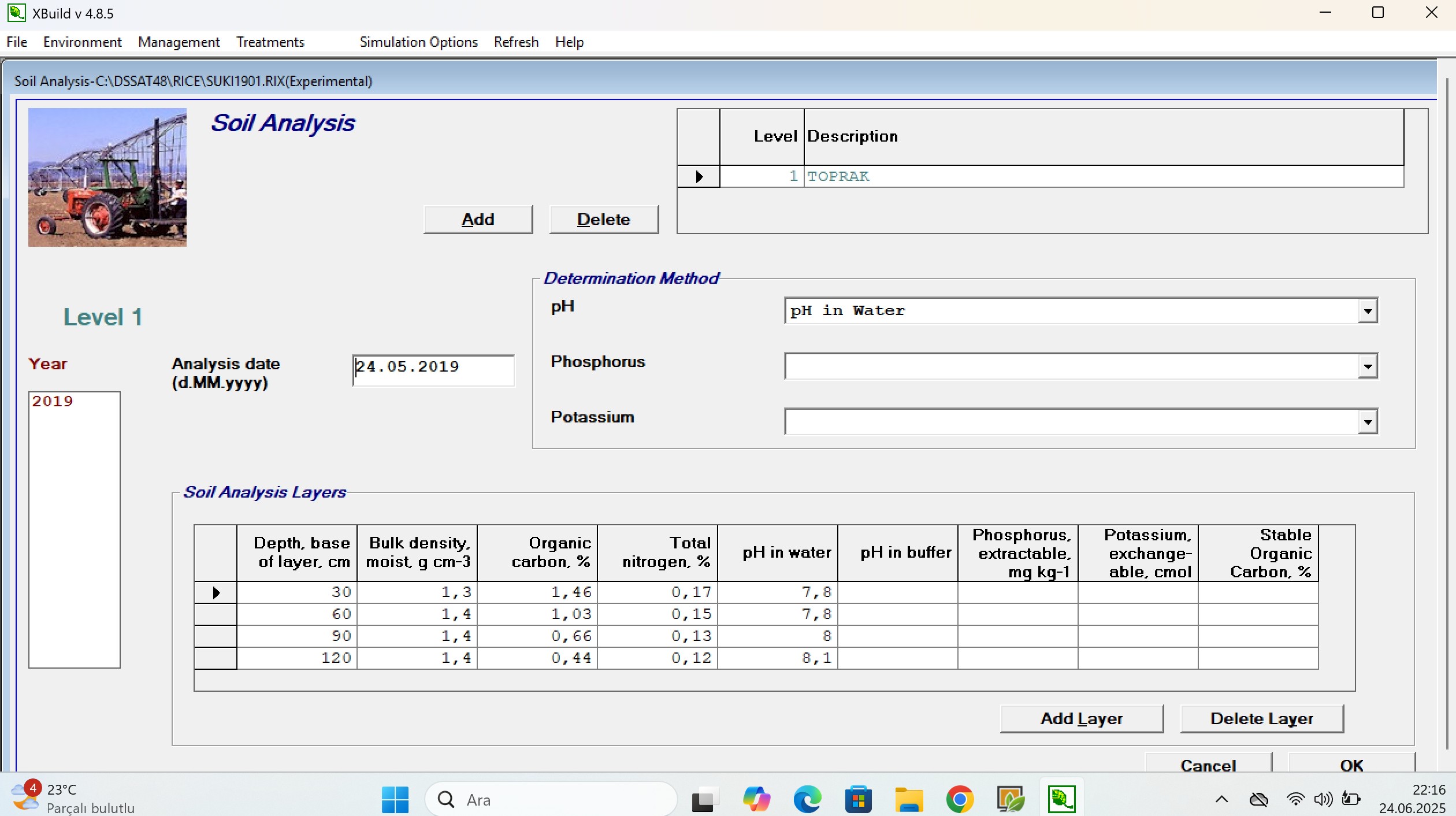

Supplement: Supplemental Information 15 — Screenshot of soil analysis section in Xbuild file in DSSAT [file peerj-14-20965-s015.jpg]
