# Supplementary material for: Assessing rice yield responses to climate change scenarios using a crop simulation model
Source: PeerJ. 2026 Mar 12;14:e20965. doi: 10.7717/peerj.20965 (PMC12989151; doi:10.7717/peerj.20965)
Supplement: Supplemental Information 16 — Explanation of the codes in the data [file peerj-14-20965-s016.docx]

**ENGLISH CODE BOOK OF DATA FILES**

1. In Overview.txt file explanation is given below:

Under MAIN GROWTH AND DEVELOPMENT VARIABLES line, variables are written as Panicle Initiation day, Anthesis day, Physiological maturity day, Leaf area index maximum and Tops weight at maturity (biomass) simulated by model and observed from fields are given for 3 run of model (I1=kpc_1.00_, I2=kpc_1.25_ and I3=kpc_1.50_)

1. In PlantGro.txt file explanation is given below:

LAID column: Leaf area index values for day of year (example: LAI value for 2019 year, 254 day is 1.69)

GWAD column: Yield value (example: Yield value for 2019 year, 254 day is 7091)

CWAD column: Biomass value (example: Biomass value for 2019 year, 254 day is 14508)

1. In RICER048_CUL.txt file explanation is given below:

Last line in txt file is the cultivar which was used in study. As in the title REKOR rice cultivar was used but we can code this with four letter. REKO was calibrated cultivar in the study. Every column is cultivar coefficients.

P1 Time period (expressed as growing degree days [GDD] in oC-d above a base temperature of 9oC) from seedling emergence during which the rice plant is not responsive to changes in photoperiod.

P2O Critical photoperiod or the longest day length (in hours) at which the development occurs at a maximum rate.

P2R Extent to which phasic development leading to panicle initiation is delayed (expressed as GDD in oC-d) for each hour increase in photoperiod above P2O.

P5 Time period in GDD oC-d) from beginning of grain filling (3 to 4 days after flowering) to physiological maturity with a base temperature of 9oC.

G1 Potential spikelet number coefficient as estimated from the number of spikelets per g of main culm dry weight (less leaf blades and sheaths plus spikes) at anthesis.

G2 Single grain weight (g) under ideal growing conditions, i.e. nonlimiting light, water, nutrients, and absence of pests and diseases.

G3 Tillering coefficient (scalar value) relative to IR64 cultivar under ideal conditions.

PHINT Phyllochron Interval (oC-d). Time interval in degree-days for each leaf-tip to appear under non-stressed conditions.

THOT Temperature (oC) above which spikelet sterility is affected by high temperature.

TCLDP Temperature (oC) below which panicle initiation is further delayed (other than P1, P2O and P2R) by low temperature.

TCLDF Temperature (oC) below which spikelet sterility is affected by low temperature.

1. SUKI1901_A.txt file explanation is given below:

LAIX: Maximum leaf area index

ADAT: Anthesis date (day of year)

MDAT: Physological maturity date

EDAT: Emergence date

CWAM: Biomass value

IDAT: Panicle initation date

- For all irrigation treatments (TRNO1: KPC_1.00_, TRNO2: KPC_1.25_, TRNO3: KPC_1.50_)

1. SUKI1901_T.txt file explanation is given below:

LAID: Leaf area index value

CHTD: Crop height value (not used for validation or calibration. Validation and calibration stages are given in the title)

**PLEASE NOTE THAT RESULTS OF THE STUDY WAS IN 2020 YEAR. IN DSSAT MODEL 2019 YEAR REFLECTS 2020 YEAR.**
